# Is Beak Morphology in Darwin’s Finches Tuned to Loading Demands?

**DOI:** 10.1371/journal.pone.0129479

**Published:** 2015-06-12

**Authors:** Joris Soons, Annelies Genbrugge, Jeffrey Podos, Dominique Adriaens, Peter Aerts, Joris Dirckx, Anthony Herrel

**Affiliations:** 1 Laboratory of Biomedical Physics, University of Antwerp, Groenenborgerlaan 171, 2020, Antwerpen, Belgium; 2 Evolutionary Morphology of Vertebrates, Ghent University, K.L. Ledeganckstraat 35, 9000, Gent, Belgium; 3 Department of Biology, 221 Morrill Science Center, University of Massachusetts, Amherst, Massachusetts, 01003, United States of America; 4 Department of Biology, University of Antwerp, Universiteitsplein 1, 2610, Antwerpen, Belgium; 5 Department of movement and sports sciences, University of Ghent, Watersportlaan 2, 9000, Gent, Belgium; 6 Département d’Ecologie et de Gestion de la Biodiversité, Museum National d’Histoire Naturelle, 57 rue Cuvier, Case postale 55, 75231, Paris, Cedex 5, France; Max Planck Institute for Evolutionary Anthropology, GERMANY

## Abstract

One of nature's premier illustrations of adaptive evolution concerns the tight correspondence in birds between beak morphology and feeding behavior. In seed-crushing birds, beaks have been suggested to evolve at least in part to avoid fracture. Yet, we know little about mechanical relationships between beak shape, stress dissipation, and fracture avoidance. This study tests these relationships for Darwin's finches, a clade of birds renowned for their diversity in beak form and function. We obtained anatomical data from micro-CT scans and dissections, which in turn informed the construction of finite element models of the bony beak and rhamphotheca. Our models offer two new insights. First, engineering safety factors are found to range between 1 and 2.5 under natural loading conditions, with the lowest safety factors being observed in species with the highest bite forces. Second, size-scaled finite element (FE) models reveal a correspondence between inferred beak loading profiles and observed feeding strategies (e.g. edge-crushing versus tip-biting), with safety factors decreasing for base-crushers biting at the beak tip. Additionally, we identify significant correlations between safety factors, keratin thickness at bite locations, and beak aspect ratio (depth versus length). These lines of evidence together suggest that beak shape indeed evolves to resist feeding forces.

## Introduction

The often tight correspondence between bird beaks and plant morphology well-illustrates the power and precision of natural selection [1]. Within bird populations, subtle variations in beak morphology can affect foraging efficiency [2], and in some cases tip the balance between survival and starvation [3]. For seed-crushing birds, however, analyses of feeding capacity require a broader view than just beak morphology *per se*. This is because a bird’s ability to crush seeds is determined mainly by bite force capacity, which in turn depends primarily on the orientation and cross sectional area of the jaw closer muscles [4–6]. These muscles, situated at the back of the head, generate crushing forces that are transferred to food by means of the upper and lower beak through a complex kinetic beak apparatus [6–11]. Beak morphology, by contrast, likely evolves to facilitate successful food manipulation and song production. However, beaks also need to avoid structural failure during loading [4]. The loading regime itself presumably varies with food manipulation strategies (e.g. tip biting versus base crushing) and may thus drive the evolution of beak shape [12].

We here test, in Darwin’s finches of the Galápagos Islands, the hypothesis that beak morphology provides structural integrity, i.e., that it evolves to resist feeding forces and avoid fracture [13]. Beaks in Darwin’s finches are known to vary broadly across and within species, and to evolve via natural selection as a response to variation in food type, food availability, and interspecific competition [3,10,14–18]. Our main approach is to develop and apply finite element (FE) models of the upper beak. FE models enable exploration of the effect of complex shape variation on stress magnitude and distribution [19–21], and allows us to draw inferences about patterns of loading during biting. We include here 13 species and incorporate data into our models for the bones, the jaw closer muscles, and the keratinous rhamphotheca that encapsulates the beak. Including the rhamphotheca in the model allows us to provide a more realistic estimate of stresses incurred during biting [22,23] that we achieved in previous studies [24,25].

The goal of this paper is twofold. First we examine how natural loadings are reflected in safety factors for a subset of species for which physiological FE models could be created (i.e. for which data on muscles could be obtained). Second, we created models of all 13 species scaled to a common surface area to muscle ratio [26]. This allows us to test whether variation in beak shape matches differences in food handling behavior. We predict that species will have beak shapes that allow them to minimize peak stresses during those feeding behaviors most commonly employed. In particular, we predict that base-crushing species should show the lowest stresses when biting at the base of the beak, that tip-biting species should show the lowest stresses during tip-biting, and that probing beaks should show higher stresses during both tip- and base crushing compared to those species specialized for these behaviors.

## Materials and Methods

### Specimen collection and CT scanning

No animals were killed for the purpose of this study. Road-killed specimens were collected during February-March of 2005 and 2006 on the main road connecting the airport on Baltra with Puerto Ayora, Santa Cruz Island. Specimens were collected under a salvage permit provided by the Galápagos National Park Service. Intact specimens were collected and preserved in a 10% aqueous formaldehyde solution for 24 h, rinsed and transferred to a 70% aqueous ethanol solution. One individual of *Geospizia fortis*, *Geospizia fuliginosa*, *Geospizia scandens*, *Platyspiza crassirostris*, *Certhidea olivacea*, and *Camarhynchus parvulus* were scanned at the UGCT scanning facility (www.ugct.ugent.be), using a micro-focus directional type X-ray tube, set at a voltage of 80 kV and a spot size of 10 mm. Specimens were mounted on a controllable rotating table (MICOS, UPR160F-AIR). For each specimen, a series of 1000 projections of 940 by 748 pixels were recorded covering 360 degrees.

Museum specimens of *Geospizia magnirostris*, *Geospizia difficilis*, *Pinaroloxias inornata*, *Cactospiza pallida*, *Camarhynchus psittacula*, *Geospiza conirostris*, and *Camarhynchus pauper* from the collections of the California Academy of Sciences maintained in a 70% aqueous ethanol solution were scanned at the Harvard CNS facility using an X-Tek XRA-002 micro-CT imaging system set at 75 kV. Specimens were mounted on a rotating table and a series of 3142 projections of 2000 by 2000 pixels covering 360 degrees was recorded. The voxel size of the scans was dependent on the specimen size and ranged from 2.96 μm for the smallest species to 47.00 μm for the larger species. As such, the bone shell of the bony core was represented by multiple voxels, and details within the bony beak were visible.

### Muscle data

Muscle data were available for five of the species collected in the field as road-killed specimens: *G*. *fortis*, *G*. *fuliginosa*, *G*. *scandens*, *C*. *olivacea* and *C*. *parvulus*; data for three additional species were derived through dissection of museum specimens from the California Academy of Sciences (*G*. *magnirostris*, *G*. *conirostris*, and *G*. *difficilis*). These species were dissected and all muscle bundles of the jaw removed individually [27]. Muscles were blotted dry and weighed on a Mettler microbalance (±0.01mg). Next, muscles were transferred individually to Petri dishes and submerged in a 30% aqueous nitric acid solution for 18 h to dissolve all connective tissue [28]. After removal of nitric acid, muscles were transferred to a 50% aqueous glycerol solution and fibers were teased apart using blunt-tipped glass needles. Thirty fibers were selected from each muscle bundle (Table 1) and drawn using a binocular scope with attached *camera lucida*. A background grid was also drawn in each image to provide an object for scaling. Drawings were scanned and fiber lengths determined using imageJ (freely available at http://rsb.info.nih.gov/ij/).

**Table 1:** Table summarizing muscle mass and fiber length data used to calculate the external forces acting on the upper beak of the different species of Darwin’s finch.

Species		MDM	MAMER	MAMEV	MAMEP	MAMOQ	MPsTSl	MPsTSm	MPsTP	MPtVl	MPtVm	MPtDl	MPtDm	MRPal	MPPtQ
*G*. *magnirostris*	mass	0.0522	0.1813	0.0451	0.0595	0.0084	0.0137	0.0624	0.0447	0.0752	0.0758	0.0568	0.022	0.0104	0.0085
	fl	3.99	2.31	2.01	2.02	2.58	1.36	1.39	4.18	2.58	2.56	2.56	2.63	2.61	2.65
*G*. *cornirostris*	mass	0.0455	0.0389	0.0178	0.0262	0.0035	0.0033	0.0157	0.0269	0.0079	0.0253	0.0240	0.0093	0.0044	0.0086
	fl	4.12	2.55	2.93	2.37	2.21	1.81	2.62	4.52	3.10	3.20	3.42	2.65	2.73	2.63
*G*. *difficilis*	mass	0.005	0.0102	0.0025	0.0044	0.0002	0.0006	0.0012	0.0064	0.0020	0.0055	0.0071	0.003	0.0014	0.0004
	fl	2.91	1.67	2.01	1.72	1.64	1.53	2.15	2.76	2.36	4.32	2.54	2.21	1.98	1.62
*G*. *fuliginosa*	mass	0.0070	0.0163	0.0035	0.0069	0.0010	0.0020		0.0020	0.0027	0.0036	0.0052	0.0032	0.0014	0.0020
	fl	2.85	2.13	1.41	1.46	2.02	1.12		1.46	2.09	1.99	1.69	2.10	2.60	2.22
*C*. *parvulus*	mass	0.0056	0.0130	0.0029	0.0065	0.0005	0.0013		0.0022	0.0044	0.0016	0.0027	0.0029	0.0012	0.0040
	fl	3.45	3.07	2.11	1.92	1.44	1.67		1.58	2.81	2.81	3.19	2.75	3.18	2.61
*C*. *olivacea*	mass	0.0033	0.0041	0.0011	0.0022		0.0007		0.0005	0.0021	0.0014	0.0008	0.0016	0.0004	0.0019
	fl	2.05	1.74	1.14	1.33		1.03		1.30	2.66	2.27	2.47	1.86	1.80	1.86
*G*. *scandens*	mass	0.015	0.0329	0.009	0.0126	0.0016	0.0071	0.0052	0.0168	0.0072	0.0062	0.0247	0.0108	0.0057	0.0042
	fl	3.89	2.52	1.89	1.98	2.64	1.22	1.22	1.54	2.99	2.96	2.70	2.75	2.60	2.30
*G*. *fortis*	mass	0.02	0.0651	0.0159	0.0217	0.0026	0.0094	0.0124	0.0283	0.0199	0.0119	0.0284	0.0333	0.0041	0.0059
	fl	3.91	1.87	1.85	1.70	2.02	1.31	2.06	3.58	2.38	2.52	2.07	1.73	2.88	3.40

Mass in gram, fiber lengths in mm. *N* = 1 for all species except *G*. *fortis* where *N* = 4. For the smaller species *C*. *olivacea*, *C*. *parvulus* and *G*. *fuliginosa* the distinction between the medial and lateral superficial m. pseudotemporalis was extremely difficult to make. As such both layers were grouped. Similarly, for the smallest species, *C*. *olivacea*, we were unable to extract the m. adductor mandibulae ossi quadrati (MAMOQ). MDM, m. depressor mandibulae; MAMER, m. adductor mandibulae externis rostralis; MAMEV, m. adductor mandibulae externus ventralis; MAMEP, m. adductor mandibulae externus profundus; MAMOQ, m. adductor mandibulae ossi quadrati; MPsTSl, m. pseudotemporalis superficialis pars lateralis; MPsTSm, m. pseudotemporalis superficialis pars medialis; MPsTP, m. pseudotemporalis profundus; MPtVl, m. pterygoideus ventralis pars lateralis; MPtVm, m. pterygoideus ventralis pars medialis; MPtDl, m. pterygoideus dorsalis pars lateralis; MPtDm, m. pterygoideus dorsalis pars medialis; MRPal, m. retractor palatine; MPPtQ, m. protractor pterygoidei et quadrati.

Based on muscle mass and fiber length, the physiological cross-sectional area of each muscle bundle was estimated assuming a muscle density of 1036kg/m^3^ [29]. Since pennate muscles were separated into their individual bundles, no additional correction for pennation angle was included. Corresponding force-generation capacities for each muscle were calculated assuming a muscle stress of 30N/cm^2^ [30]. As the external adductor and pseudotemporalis muscles act only indirectly on the upper mandible [6,9,10,31], the component of the muscle force transferred to the upper mandible was calculated taking into account the position of the muscles and their angles relative to the jugal bone (which transfers the forces from the lower jaw and the quadrate to the upper beak by pulling the beak downward when quadrate is rotated backward during jaw closing). The pterygoid muscle bundles act directly on the upper mandible [6,9,10,31], and muscle forces are directly transmitted through the pterygoid/palatine complex. The combined muscle forces applied to the model are shown in Table 2.

**Table 2:** Von Mises stress for different loading conditions (LC) for 13 Darwin’s finches (PB: physiological base biting; PT: physiological tip biting; FB: base biting scaled to *fortis*; FT: tip biting scaled to *fortis*).

Species	LC	Fj	Fp	vM1	vM2	vM3	GV1	GV2	GV3	F	V_bone_	V_ker_
*G*. *fortis*	PB	11.9	10.9	21	19	29	0.83	0.98	0.81	30.4	104	128
*G*. *fortis*	PT	11.9	10.9	47	36	41	0.78	0.95	0.82	21.5	104	128
*G*. *magnirostris*	PB	23.0	21.9	35	24	40	0.86	0.97	0.77	57.9	232	195
*G*. *magnirostris*	PT	23.0	21.9	66	35	46	0.62	0.98	0.81	41.6	232	195
*G*. *magnirostris*	FB	11.9	10.9	22	16	26	0.86	0.97	0.77	29.3	157	131
*G*. *magnirostris*	FT	11.9	10.9	43	23	30	0.62	0.98	0.81	20.9	157	131
*G*. *fuliginosa*	PB	2.2	3.2	18	15	18	0.74	0.93	0.85	7.1	24	35
*G*. *fuliginosa*	PT	2.2	3.2	24	28	27	0.60	0.88	0.84	4.6	24	35
*G*. *fuliginosa*	FB	11.9	10.9	29	23	28	0.74	0.93	0.85	29.2	115	164
*G*. *fuliginosa*	FT	11.9	10.9	35	41	42	0.60	0.88	0.84	19.0	115	164
*G*. *conirostris*	PB	5.4	5.3	16	25	42	0.86	0.85	0.76	15.3	76	82
*G*. *conirostris*	PT	5.4	5.3	25	40	47	0.98	0.83	0.70	10.4	76	82
*G*. *conirostris*	FB	11.9	10.9	25	33	67	0.86	0.85	0.76	32.3	112	121
*G*. *conirostris*	FT	11.9	10.9	45	63	67	0.98	0.83	0.70	18.8	112	121
*G*. *scandens*	PB	3.2	4.5	20	15	23	0.67	0.92	0.74	8.9	59	56
*G*. *scandens*	PT	3.2	4.5	17	33	35	0.88	0.95	0.82	6.1	59	56
*G*. *scandens*	FB	11.9	10.9	40	28	43	0.67	0.92	0.74	26.2	126	119
*G*. *scandens*	FT	11.9	10.9	30	60	63	0.88	0.95	0.82	17.9	126	119
*G*. *difficilis*	PB	1.5	1.6	14	12	21	0.87	1.00	0.97	3.6	22	30
*G*. *difficilis*	PT	1.5	1.6	18	19	18	0.73	0.98	0.98	2.4	22	30
*G*. *difficilis*	FB	11.9	10.9	38	24	51	0.87	1.00	0.97	26.1	86	116
*G*. *difficilis*	FT	11.9	10.9	50	56	53	0.73	0.98	0.98	16.7	86	116
*P*. *inornata*	FB	11.9	10.9	55	32	59	0.66	0.95	0.85	26.2	109	96
*P*. *inornata*	FT	11.9	10.9	76	67	61	0.71	1.00	0.83	13.8	109	96
*C*. *olivacea*	PB	0.9	1.0	23	31	45	0.50	0.84	0.81	2.0	9	9
*C*. *olivacea*	PT	0.9	1.0	23	32	22	1.00	1.00	0.94	1.0	9	9
*C*. *olivacea*	FB	11.9	10.9	58	66	100	0.50	0.84	0.81	24.4	99	100
*C*. *olivacea*	FT	11.9	10.9	58	78	59	1.00	1.00	0.94	12.3	99	100
*C*. *pallida*	FB	11.9	10.9	59	33	80	0.77	0.71	0.88	25.4	68	101
*C*. *pallida*	FT	11.9	10.9	38	46	64	0.67	0.87	0.89	15.1	68	101
*C*. *parvulus*	PB	1.3	1.3	22	12	20	0.57	0.94	0.61	3.3	11	18
*C*. *parvulus*	PT	1.3	1.3	13	19	25	0.66	0.82	0.58	2.2	11	18
*C*. *parvulus*	FB	11.9	10.9	47	26	43	0.57	0.94	0.61	29.6	88	143
*C*. *parvulus*	FT	11.9	10.9	29	41	55	0.66	0.82	0.58	19.5	88	143
*C*. *pauper*	FB	11.9	10.9	23	17	37	0.60	0.94	0.52	31.2	118	163
*C*. *pauper*	FT	11.9	10.9	19	25	35	0.82	0.99	0.75	19.4	118	163
*C*. *psittacula*	FB	11.9	10.9	39	24	33	0.46	0.88	0.90	30.5	93	127
*C*. *psittacula*	FT	11.9	10.9	25	28	35	0.71	0.63	0.90	21.9	93	127
*P*. *crassirostris*	FB	11.9	10.9	21	21	31	0.84	0.93	0.81	32.1	138	152
*P*. *crassirostris*	FT	11.9	10.9	25	32	43	0.82	0.97	0.90	21.7	138	152

**Fig 1:**
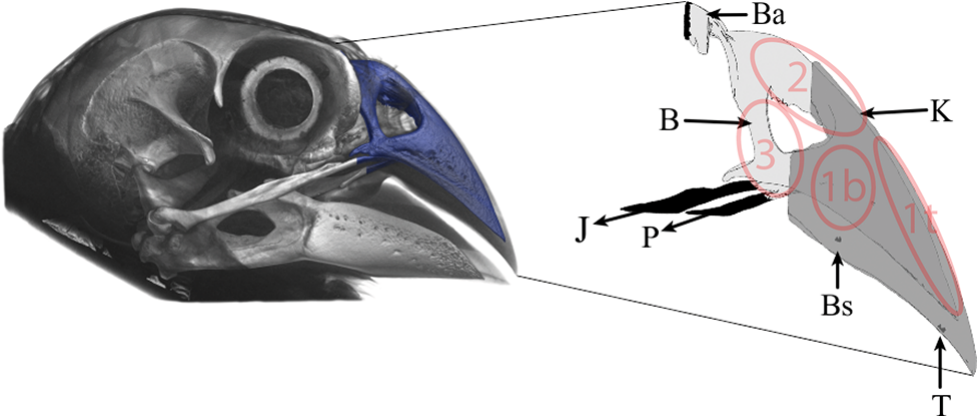
Schematic representation of our multi-layered (bone: B, keratin: K) finite element modeling approach, for the medium ground finch *Geospiza fortis*. Bending area (Ba) and bite position (base: Bs, or tip: T) were constrained in our models for translation and rotation, and muscle forces were applied in our models via the jugal (J) and palatine (P) jaw bones (black elements are constrained). Locations of vM Stress recordings are indicated with transparant ellipses (1b: on top of bone, near base bite position; 1t: on top of bone near tip bite position; 2: on top of the beak near the nasal hinge; 3: nasal bone).

Model input forces (= muscle forces) for jugal (Fj) and palatine (Fp) are given in N. von Mises stresses are given in MPa for three positions indicated in Fig 1 (vM1: on top of bone, near bite position; vM2: on top of the nasal hinge; vM3: nasal bone ipsilateral side for base biting, both sides for tip biting). Gray values in CT-stack for the same positions are given (0 black, 1 white). The resulting (model) biting force (*F*)is given in N; the volume of keratin and bone (*V*_*ker*_ and *V*_*bone*_) are given in *mm* ^*3*^.

### Segmentation and FE modelling

The segmentation of the bony core and the keratin layer (Amira 4.1 64-bit version, TGS systems) was done for all species. The tetrahedral grid construction [32], the assigned material properties (homogenuous and isotropic linear elastic with E_keratin_ = 1.7 GPa and E_bone_ = 7.3 GPa) [22] and the boundary conditions are similar to those reported previously [22–24, 33]. Convergence testing on the results, namely the von Mises (vM) stresses and the bite forces, was conducted so they change less than 5% when doubling mesh size. The final FE grids of approximately 2–3 million 4-noded linear tetrahedral elements were obtained. Consequently, multiple layers of elements were available to model the thin bony walls.

For every specimen, a FE model for base and tip loading was established. In this study we were only interested in the stresses on the upper beak, and as such elements were fully constrained (in all 6 degrees of freedom) at the back of the beak at the neurocranium. Hinging of the upper beak is possible due to bending of the thin bone (BA in Fig 1). In order to avoid singularities around point constraints, we constrained elements (in x, y and z direction, no rotation) at the ventral surface of the rhamphotheca simulating the bite constraint (T and Bs in Fig 1); the calculated reaction force of these elements is the bite force which is of equal magnitude yet of opposite sign as the seed reaction force. Bite positions were estimated using *in vivo* observations of animals cracking or manipulating seeds in the field [13]. Elements were constrained for a central tip bite close to the tip of the bony core, as birds are never seen to bite at the very tip in the wild (T in Fig 1). For an unilateral base bite we used a point at one fourth of the total length of the rhamphotheca, close to the edge of the bony core (Bs in Fig 1), hence creating homologous bite points across species. The available muscle forces (Table 2) estimated through dissection were applied to rigid elements at the end of the jugal and pterygoid bones in the direction of these bones (J and P in Fig 1). This resulted in physiological FE models, for both base and tip loading, for eight species (16 models). In addition we developed scaled FE models for all 13 species. We therefore measured the beak surface of all beaks and scaled them in such a way that they have the same surface area as *G*. *fortis*. Next we applied the muscle force of *G*. *fortis* to these size-scaled beaks. *G*. *fortis* was chosen because we had muscle data and scans available for multiple individuals of this species [27], but we could have picked one of the other 7 finches. As such, the effects of size are eliminated and the effect of beak shape variation on the mechanical behavior of the beak can be isolated for the 13 species of Darwin’s finches included here [26].

**Fig 2:**
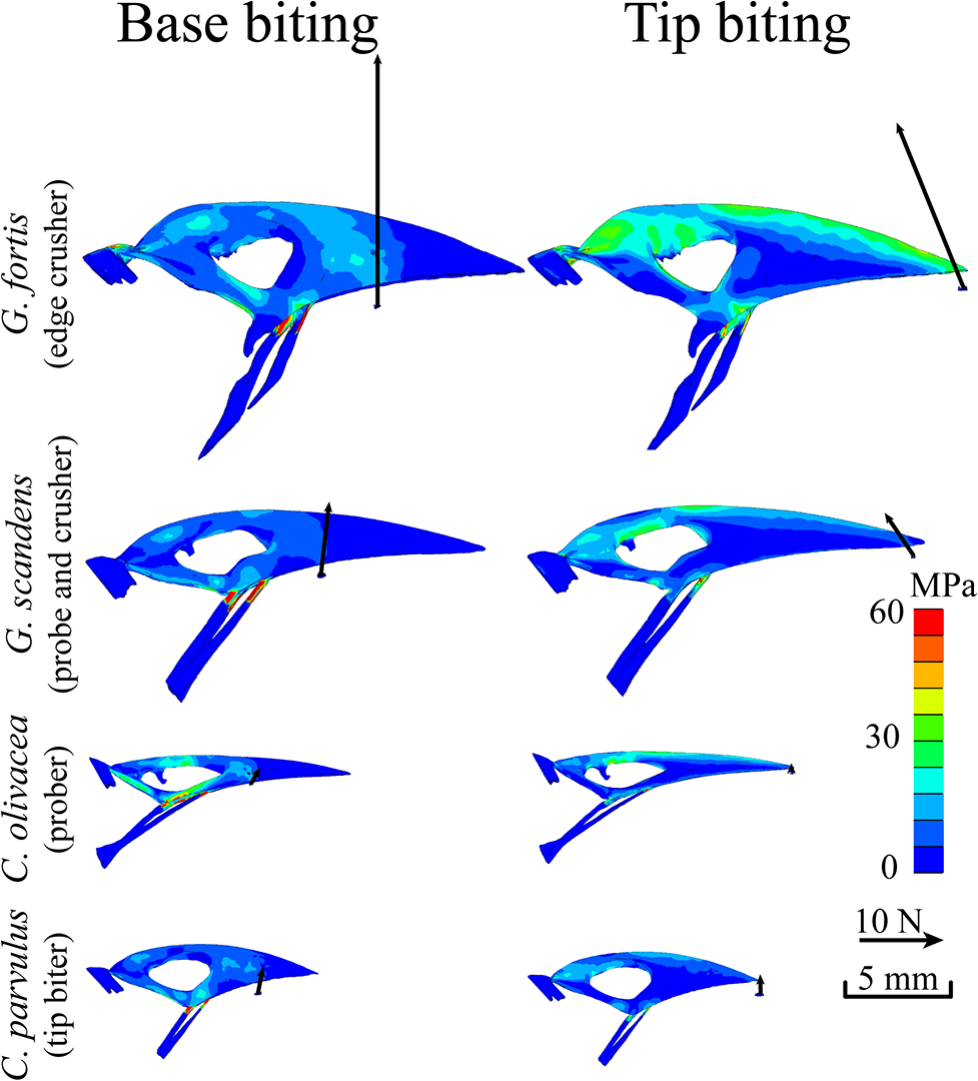
Physiological Finite Element Model results, side view, for 4 selected Darwin’s finch species known to use their beaks in different ways during feeding [10, 49]: *G*. *fortis* (base crushing beak), *G*. *scandens* (probing and crushing beak), *C*. *olivacea* (probing beak), and *C*. *parvulus* (tip biting beak). Results are shown for both base (1^st^ column) and tip (2^nd^ column) biting simulations. Arrows indicate the location and magnitude of the calculated seed reaction forces. Warmer colors represent higher von Mises stresses. Additional FE models for the eight finches for which muscle data were available are presented in S2 and S3 Figs.

**Fig 3:**
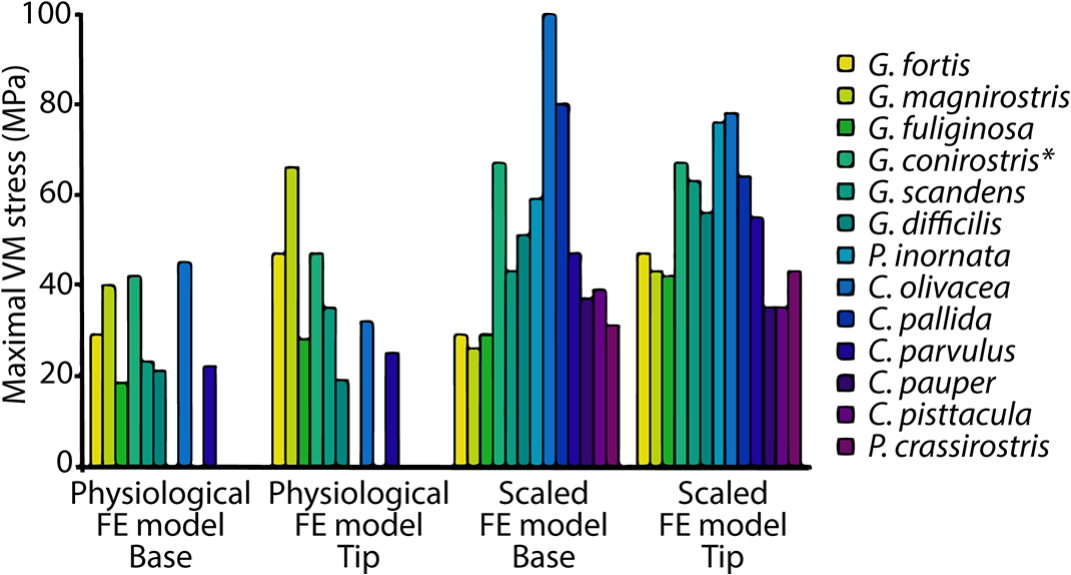
Peak vM stress for physiological FE models (tip and base loading, eight species with available muscle data) and for scaled FE models (tip and base loading, scaled to same size as *G*. *fortis* and with the same muscle forces) for thirteen species of Darwin’s finch (* = juvenile).

**Fig 4:**
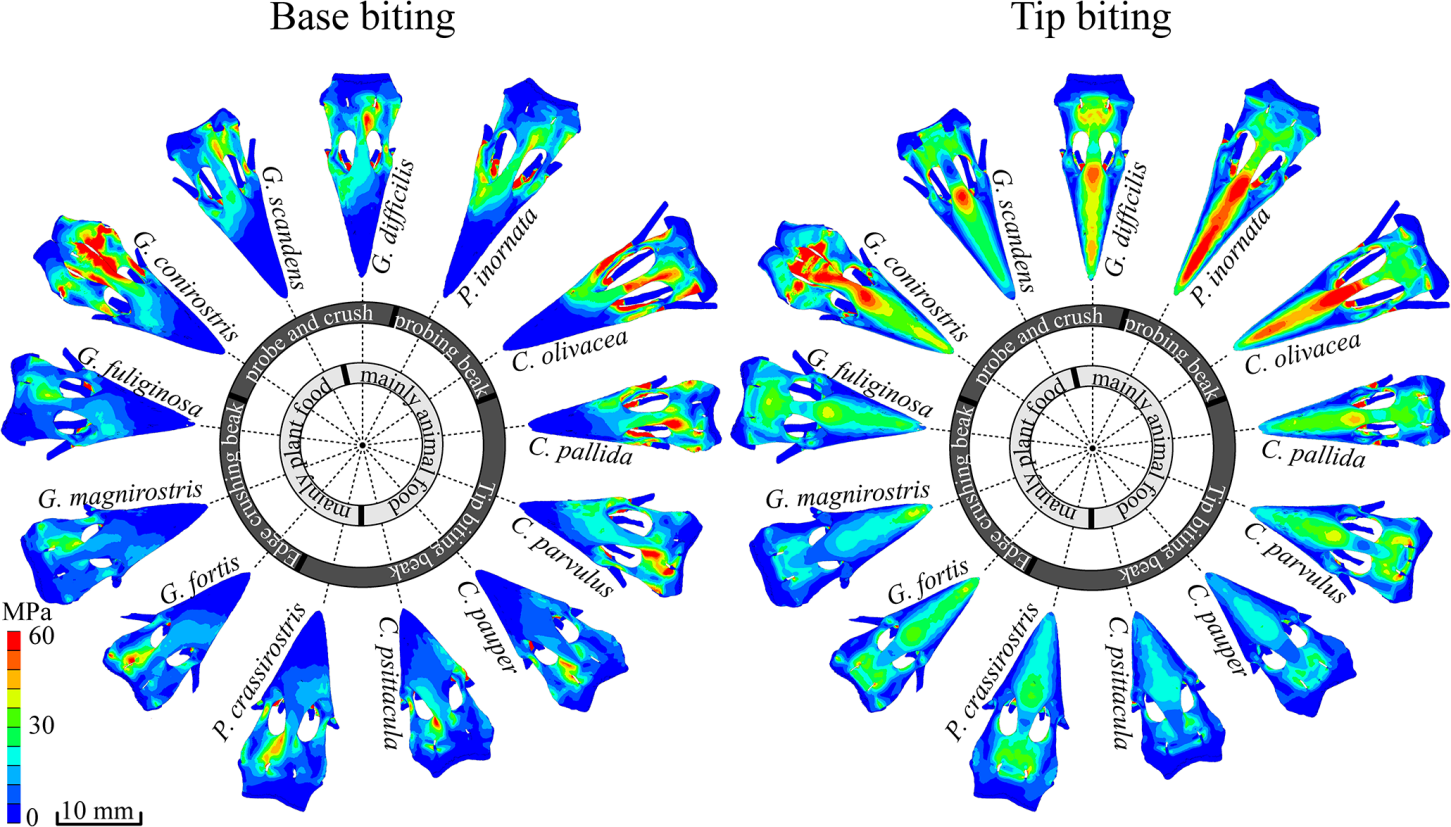
Top view of scaled FE models of the upper beaks of 13 species of Darwin’s finches. All beaks were scaled to same size and muscle force as *G*. *fortis*, with stresses calculated for both base (left) and tip (right) biting. Warmer colors represent higher vM stresses. Note how stresses are lower during the behaviors typically employed by each species, with base crushers showing the lowest stress values during base-loading and tip crushers during tip-loading. Species with probing beaks show generally high stresses under both loading conditions. Top and side views of scaled FE models are presented in S4 and S5 Figs.

Bite force and maximum vM stress were obtained for all 40 FE models (Table 2): 16 physiological models (tip and base loading for 8 beaks with available muscle data) and 24 models (tip and base loading for all 13 beaks size-scaled to *G*. *fortis*, minus *G*. *fortis*, see Table 2). Maximum vM stress was taken according to Saint-Venant's Principle over three different locations displaying the highest stresses on the bony core, away from areas influenced by model constraints (Figs 1 and 2, Table 2). We used 98^th^ percentile to select maximum vM stresses presented in Fig 3 and Table 2. In these locations, the gray values of the corresponding CT images were also recorded. It has been shown [34–36] that the strength of bone increases with density. Scan parameters were optimized for each CT scan and no calibration was performed, making it impossible to obtain the density directly from these gray values. Consequently, gray values were transferred to a qualitative linear scale, where 0 represents the least dense bone in the sample and 1 the densest. vM stresses on the keratin layer were not taken into account and are not displayed in Figs 2 and 4. Nevertheless, a correct modeling of the rhamphotheca is important since it has a significant influence on the stress distribution and magnitude in the bony core [23].

During base loading vM stresses are not recorded for the contralateral side of the nasal bone, because a bird could decrease this stress by reducing the bite force at that side. As a consequence, the resulting reaction force on the contralateral side will be lower. It is, however, very complex to introduce this bite force tuning into our models and as such is ignored here. To quantify a birds’ risk of beak fracture during biting, we calculated safety factors for each species’ beak, by dividing bone strength by peak stress. Values for bone strength were obtained using the linear relationship of Fyhrie and Vashishth, bone strength = 0.0061 E_Bone_ [37]. In this study we used 45 MPa for a experimentally obtained Young’s modulus of 7.3 GPa for finch beak bone [22,23]. Finally keratin thickness on top and bottom of the beak, and beak dimensions (depth and width relative to length) were measured and are reported for four functional groups (Table 3), and Pearson’s correlations (with Bonferroni correction) of these values compared to the SFs are shown in Table 4.

**Table 3:** Characterization of keratin thickness and beak dimensions (relative to beak length) in the different functional groups.

	bottom keratin thickness / beak length	top keratin thickness / beak length	beak depth / beak length	beak width / beak length
Crush (N = 3)	0.095 ± 0.015	0.023 ± 0.005	0.22 ± 0.03	0.406 ± 0.010
Probe and Base (N = 3)	0.067 ± 0.007	0.0205 ± 0.0013	0.161 ± 0.013	0.35 ± 0.02
Probe (N = 2)	0.029 ± 0.005	0.018 ± 0.007	0.120 ± 0.006	0.304 ± 0.003
Tip (N = 5)	0.08 ± 0.03	0.035 ± 0.011	0.22 ± 0.05	0.44 ± 0.07

Table entries are means ± standard deviations.

**Table 4:** Pearson correlation and *p*-values between beak dimensions corrected for beak length and safety factors extracted from the different models.

	bottom keratin thickness / beak length	top keratin thickness / beak length	beak depth / beak length	beak width / beak length
safety factor base biting	**0.80** (*p* = 0.001)	0.25 (*p* = 0.40)	**0.71** (*p* = 0.006)	**0.68** (*p* = 0.011)
safety factor tip biting	**0.90** (*p* < 0.001)	0.65 (*p* = 0.015)	**0.88** (*p* < 0.001)	**0.88** (*p* < 0.001)

Bold values illustrate significant correlations (p<0.05/4, Bonferroni correction) between safety factors and anatomical features.

## Results

The calculated von Mises stresses for the physiological boundary conditions in different Darwin’s finches are presented in Figs 2, 3, S2 and S3 (see also Table 2). The force vectors representing bite forces (Figs 2, S2 and S3) correspond well with field-measured *in vivo* bite forces (Table 5) [4, 5, 25]. Note, however, that *in vivo* bite forces reported here differ proportionally from those reported previously [25], as in that study the bite forces were not corrected for the lever arms of the bite force set-up. Among finches that crush seeds at the base of their beaks, safety factors varied between 0.7 and 2.5 (Table 5). Moreover, safety factors during natural loading regimes (i.e. base biting) generally decrease as bite force increases. Importantly, safety factors are lower during tip loading behaviors suggesting that beaks in these species are not optimized for tip biting. For other species, safety factors varied between 1 and 2.3 and were generally more similar between the two loading conditions.

**Table 5:** *In vivo* measured bite force (6 species, means ± standard deviations, N = number of specimens) compared with the model bite force and the model safety factors (SF).

	behavior	Measured force at base (N)	Measured force at tip (N)	Model force at base (N)	Model force at tip (N)	Model base biting SF	Model tip biting SF
***G*. *fortis***	Base crush	23 ± 9 (*N* = 382)	19 ± 7 (*N* = 382)	30	22	1.6	1.0
***G*. *magnirostris* (*N* = 29)**	Base crush	65 ± 17 (*N* = 29)	44 ± 10 (*N* = 29)	58	42	1.1	0.7
***G*. *fuliginosa* (*N* = 115)**	Base crsuh	5.5 ± 1.9 (*N* = 115)	4.6 ± 1.6 (*N* = 115)	7.1	4.6	2.5	1.6
*G*. conirostris (*)	Probe & crush			15	10	1.1	1.0
***G*. *scandens* (*N* = 64)**	Probe & crush	10 ± 3 (*N* = 64)	7 ± 3 (*N* = 64)	8.9	6.1	2.0	1.3
***G*. *difficilis***	Probe & crush			3.6	2.4	2.1	2.3
***C*. *olivacea* (*N* = 18)**	Probe	2.0 ± 0.5 (*N* = 18)	1.2 ± 0.4 (*N* = 18)	2.0	1.0	1.0	1.4
***C*. *parvulus* (*N* = 29)**	Tip crush	5.6 ± 1.3 (*N* = 29)	4.2 ± 1.2 (*N* = 29)	3.4	2.2	2.1	1.8

(* Juvenile data)

Density variation in bone can be expected and might result in a locally higher strength at high stress locations as suggested by our preliminary data. Indeed, the gray values (Table 2) at high vM stress locations were all higher than 0.5 (0 indicates the least dense bone in the sample, 1 indicates the densest bone). For these natural loading conditions, higher relative bone densities are thus observed at locations with higher stresses which can lead to increased safety factors.

The von Mises’ (vM) stresses for the 13 size-scaled models are presented in Figs 3, 4, S4 and S5 (also see Table 2). The maximum vM stress in the size-scaled models differs broadly across different species (Table 2). For example, the beaks of *C*. *olivacea* and *P*. *inornata* in our scaled models show highest peak vM stresses of up to 100 MPa, indicating that these shapes are not suited to withstand loading regimes similar to those observed for *G*. *fortis*. The deep and wide seed crushing beaks of *G*. *magnirostris*, *G*. *fortis* and *G*. *fulignosa*, show lower stresses under base loading (below 30 MPa) than under tip loading, consistent with these birds’ emphasis on base biting strategies in nature and again in accordance with our predictions. One can also observe moderate peak stresses for the tip biting beaks (*P*. *crassirostris*, *C*. *pauper*, *C*. *psittacula*, except *C*. *parvulus*), ranging from 31 MPa to 39 MPa. The probe and crush beaks (*G*. *scandens*, *G*. *difficilis*) show higher stresses, however, ranging from 43 to 51 MPa. Finally, comparatively low maximum stresses are found during tip loading for species that use the tips of their beak during foraging and have beaks with high curvature, as predicted (e.g. *Camarhynchus pauper* and *C*. *psittacula*, 35 MPa). The crushing beaks (*G*. *fortis*, *G*. *fuliginosa*, *G*. *magnirostris*) on the other hand have higher stresses (42 to 47 MPa, SF = ±1) under these loading conditions. The main exceptions to the otherwise rather precise tuning of beak strength and feeding mode concerns relatively high stresses observed for *G*. *conirostris* (67 MPa), *C*. *parvulus* (55 MPa), and *Cactospiza pallida* (80 MPa) under different loading regimes.

The rhamphotheca was thinnest in the probers (Table 3). Base crushers have a thicker keratin layer on the bottom of the beak than tip biters, and tip biters have thicker keratin on the top of the beak. The size scaled models show a strong correlation between the beak safety factor and the thickness of the keratin at the base of the beak (Table 4). It is also interesting to note that base and tip biters seem to have similar aspect ratios of the beak (depth and width relative to length), and that these ratios are lower in the probers (Table 3). Moreover, we observe a strong and positive correlation between aspect ratio and beak safety factor (Table 4).

## Discussion

For all species, the estimated maximum vM stresses, particularly near the nasal hinge, were 4–44% lower than maximum vM stresses calculated in prior models that did not take into account the keratinous rhamphotheca [24]. An earlier study on *Padda oryzivora* [23] showed that changing the bone modulus value also had a linear effect, albeit small, on safety factor values (SFs changed from 2.5 to 3.0 if bone modulus changed from 6.7 to 7.9 GPa). Also, variation in the keratin modulus within its measured interval (Eker = 1.3 till 2.1 GPa) had a little effect on SF. However, if we decreased the keratin modulus below this, a profound effect was observed (for Eker of 1.7 GPa the SF = 2.8, while for Eker = 0.5 GPa the SF = 1.8), indicating the importance of incorporating the keratin layer in the models. Models that integrate information about multiple layers ideally consider well-defined material properties for each constituent material. These parameters were obtained on a different species of finch (*Padda oryzivora*) since experiments cannot be conducted with Darwin’s finches due to the protected status of these birds. Stress regimes for multi-layered beaks using these experimentally-obtained elastic moduli of keratin and bone had been modeled with success previously for *Padda oryzivora* and then validated using digital speckle pattern interferometry [22,23].

Keratin itself is also a multi-layered structure [38,39] that typically shows an anisotropic mechanical behavior, with preferential directions of failure depending on cell orientation [38]. The mechanical behavior of keratin also depends on its hydration state [22,40]. However, in living animals the hydration state of the keratin is controlled to the underlying living tissues (epidermis and dermis). In birds specifically, the beak consists of four distinct layers: a layer of dead epidermal cells (stratum corneum), a series of living epidermal cells, the dermis, and finally the bony beak [41]. Keratin cells typically orient such that they are aligned with the principal deformation [41]. In contrast to bone, which is a stiff mineralized tissue prone to fracture, keratin often shows local failure which does not penetrate the entire structure due to the organization of the cells in the different layers of the structure. Moreover, keratin is abrasion resistant and worn layers are shed and renewed by the addition of new layers of the stratum corneum through deposition from the living epidermal cells. As such the keratinous rhamphotheca is a continuously growing structure where damaged cells are shed and replaced. Consequently, we here decided to focus on the mechanical behavior of the underlying bony beak while taking into account the mechanical behavior of the keratinous rhamphoteca.

We found that the beaks of different finch species operate within a range of safety factors between 0.7 and 2.5 (Table 5). From an engineering perspective this range cannot be regarded as narrow, although from a biological perspective it is rather narrow if one takes into account the large variation finches express in bite forces (1N to 65N), beak sizes (1cm to 2cm), shapes, and biological uses. The *in vivo* realistic range of applicable safety factors is likely even narrower than this, given that the 0.7 value was calculated for a behavior rarely observed in nature: tip-biting in the largest species, *G*. *magnirostris*. A safety factor lower than 1 implies that the beak would break if the bird would use its full muscle strength while biting at the tip. This is, however, a biting strategy rarely observed in this base-biting bird (note that this species displays a safety factor of 1.1 for base loading). Moreover, bone remodeling could further increase safety factors by decreasing vM stresses in highly loaded areas, a statement supported by the higher grayscale values in these regions. In future modeling, density dependent elastic moduli and strength could be used to provide further insights in this material tuning. Nevertheless, calculated safety factors imply that beaks can withstand loading under normal conditions, although the exceptional jaw muscle hypertrophy of the ground finches [27], particularly *G*. *magnirostris* (S1 Fig), introduces risks to the beak’s structural integrity during tip biting.

As a further caveat, the magnitude of the safety factors given here should be interpreted as relative only when comparing different species, since our measures for the Young’s modulus were based on data for a different species (*P*. *oryzivora*). Moreover, we used the linear approximation of Fyhrie and Vashishth [37]. Indeed, a wide range of strengths for denser bone, ranging from 106 to 224 MPa [42–44] is available in literature and Darwin’s finches could potentially have evolved denser bone with a higher strength. Moreover, stress magnitudes are sensitive to changes in material properties [23,45–48]. Another assumption we make in calculating the safety factors is that the beaks of Darwin’s finches have similar material properties as those measured for *P*. *oryzivora*. We are, however, unable to test this directly since the preservation of the tissues can have a major impact on the mechanical properties of bone and keratin. In addition, freshly killed animals cannot be obtained.

A unique advantage of FE models, applied in our study, is that they can be size-scaled, thus allowing a size-independent view on the structural merits of different beak shapes. In our set of FE models, for which we scaled the beaks of all Darwin’s finches to the same size and applied identical muscle forces [26], our primary result is that maximum vM stresses differ broadly across different species, in ways that align with the different species feeding strategies (Table 2). For example, the beaks of *C*. *olivacea* and *P*. *inornata* show highest peak vM stresses in our models (up to 100 MPa). This suggests that their beak shape is not suited to crack very hard seeds. Indeed, these birds use their beaks almost exclusively to capture insects, rather than to crush or manipulate hard objects [49].

The high stresses for *G*. *conirostris* observed in our models might be explained by the fact that the geometry included in our study was that from a juvenile, while the applied muscle forces on this juvenile geometry were those from an adult. Likely, ontogenetic changes in shape and ossification take place to optimize the beak for adult loading conditions [50] as previously demonstrated for *G*. *fortis*. Results for *C*. *parvulus* and *C*. *pallida* remain puzzling, but suggest that these species may be functionally constrained in the use of their beak when applying large muscle forces.

We observed a thicker rhamphotheca in base crushing and tip-biting birds compared to birds that probe and bite, as well as dedicated probers (Table 3). Moreover, the keratin thickness at the bottom of the beak was strongly correlated to the beak safety factor indicating that thicker keratin helps protect the beak and increase its safety factor (Table 4). Interestingly, the thicker top keratin for tip biters matches the patterns of stress generated during tip loading, suggesting that in both ecotypes keratin is an important part of the stress mitigation strategy. In addition, positive and high correlations between beak aspect ratio and beak safety factor indicate that birds with relatively deeper and wider beaks have higher safety factors (Table 4) and are thus better equipped to withstand loading due to biting. This provides evidence that beak shape affects the risk of failure.

In summary, our FE models demonstrate that beak shapes are generally well-suited for mitigating risk of fracture in accordance with a species’ predominant feeding habitat. Understanding how beaks evolve to reduce risk of fracture may help to explain patterns of selection on beak size and shape in natural populations, and ultimately should be considered as one of the axes of adaptation and specialization in the Darwin’s finch radiation.

## Data availability

Data is available from biomesh.org.

## Supporting Information

S1 Fig Muscle mass and tip bite force compared to the body mass of finches. Note how *G*. *magnirostris* and *G*. *fortis* have an exceptionally high muscle mass and bite force for their size.
(DOCX)

S2 Fig Top and side view for physiological FE models of upper beak during base biting for 8 Darwin finches. (DOCX)

S3 Fig Top and side view for physiological FE models of upper beak during tip biting for 8 Darwin finches. (DOCX)

S4 Fig Top and side view for scaled FE models of upper beak during base biting for 13 Darwin finches. (DOCX)

S5 Fig Top and side view for scaled FE models of upper beak during tip biting for 13 Darwin finches. (DOCX)
