# Supplementary material for: Is Beak Morphology in Darwin’s Finches Tuned to Loading Demands?
Source: PLoS One. 2015 Jun 12;10(6):e0129479. doi: 10.1371/journal.pone.0129479 (PMC4466803; doi:10.1371/journal.pone.0129479)
Supplement: S1 Fig — Note how G. magnirostris and G. fortis have an exceptionally high muscle mass and bite force for their size. (DOCX) [file pone.0129479.s001.docx]

**S1 Fig.** Muscle mass and tip bite force compared to the body mass of finches. Note how *G. magnirostris* and *G. fortis* have an exceptionally high muscle mass and bite force for their size.
