# Supplementary material for: Is Beak Morphology in Darwin’s Finches Tuned to Loading Demands?
Source: PLoS One. 2015 Jun 12;10(6):e0129479. doi: 10.1371/journal.pone.0129479 (PMC4466803; doi:10.1371/journal.pone.0129479)
Supplement: S3 Fig — (DOCX) [file pone.0129479.s003.docx]

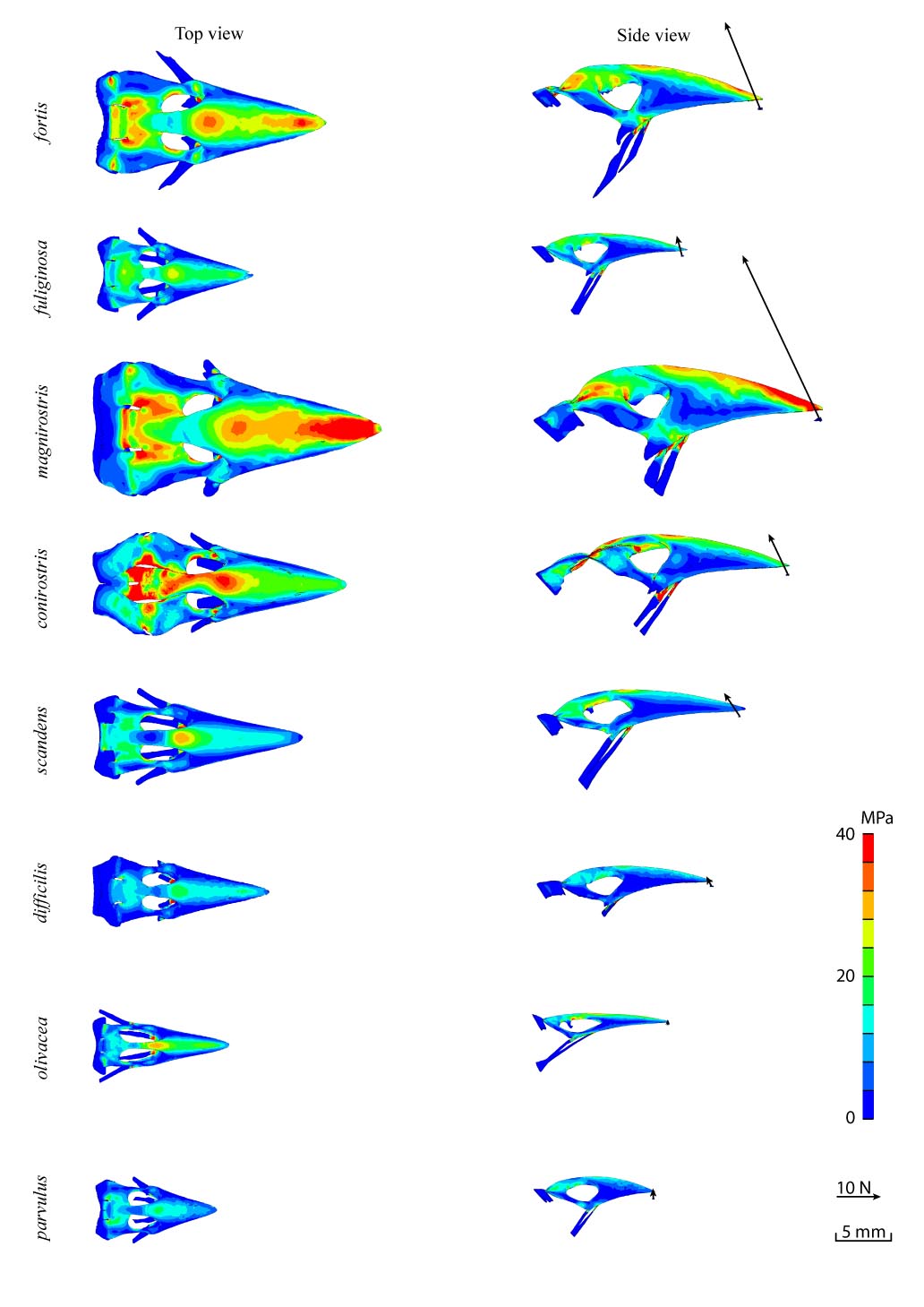


**S3 Fig.:** Top and side view for physiological FE models of upper beak during tip biting for 8 Darwin finches.
