# Supplementary figures and images for: Is Beak Morphology in Darwin’s Finches Tuned to Loading Demands?
Source: PLoS One. 2015 Jun 12;10(6):e0129479. doi: 10.1371/journal.pone.0129479 (PMC4466803; doi:10.1371/journal.pone.0129479)

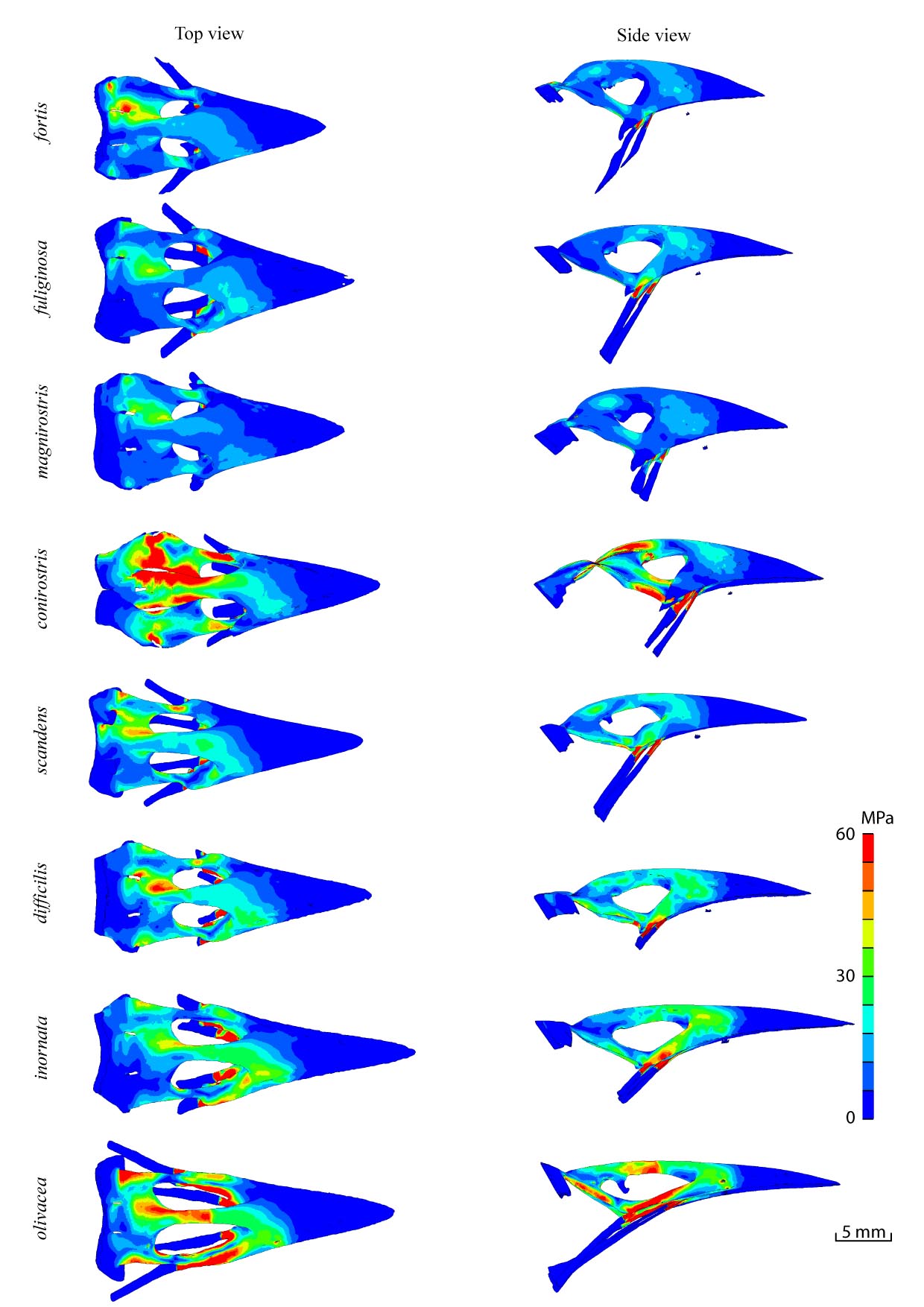


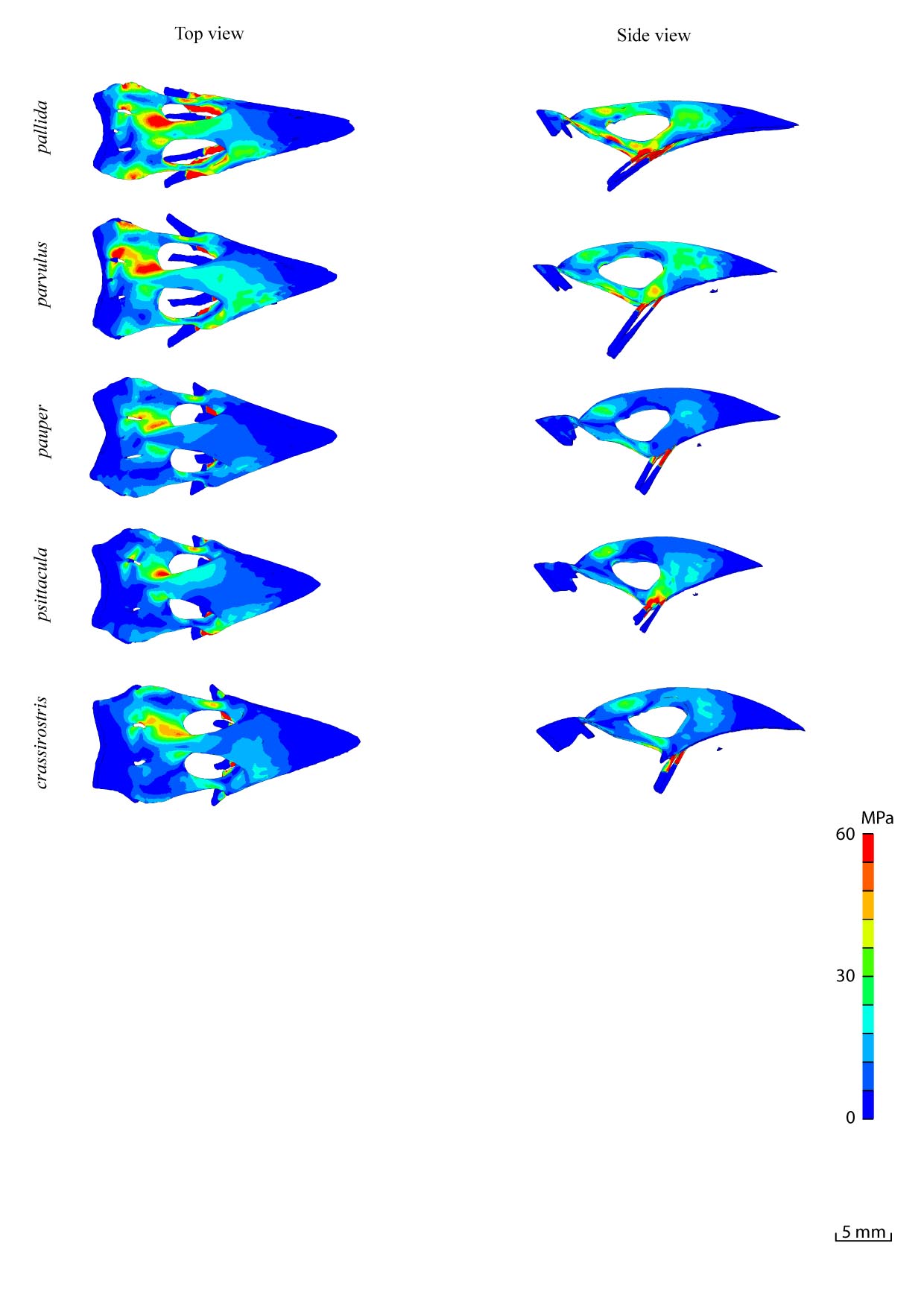


**S4 Fig.:** Top and side view for scaled FE models of upper beak during base biting for 13 Darwin finches.

Supplement: S4 Fig — (DOCX) [file pone.0129479.s004.docx]

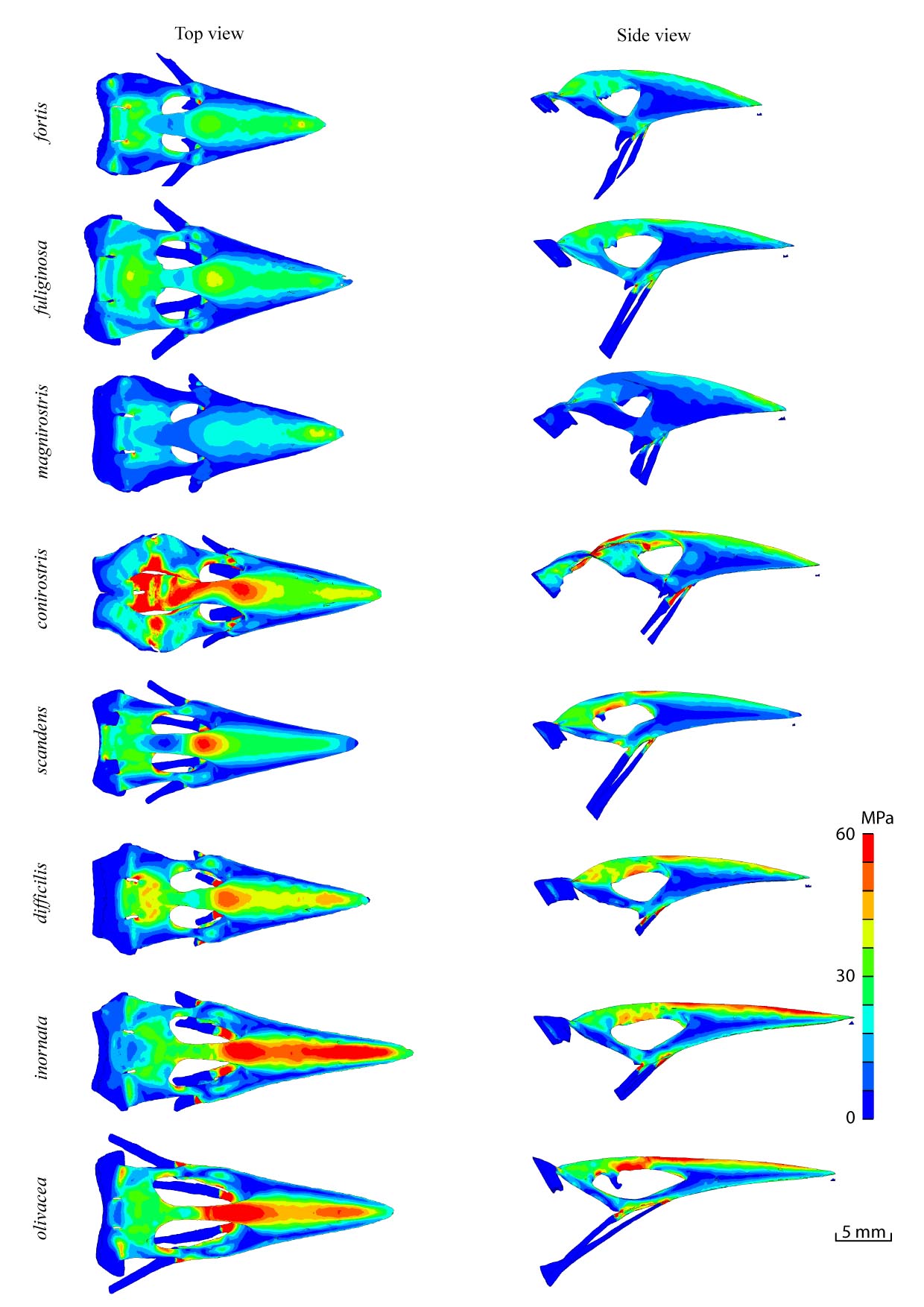


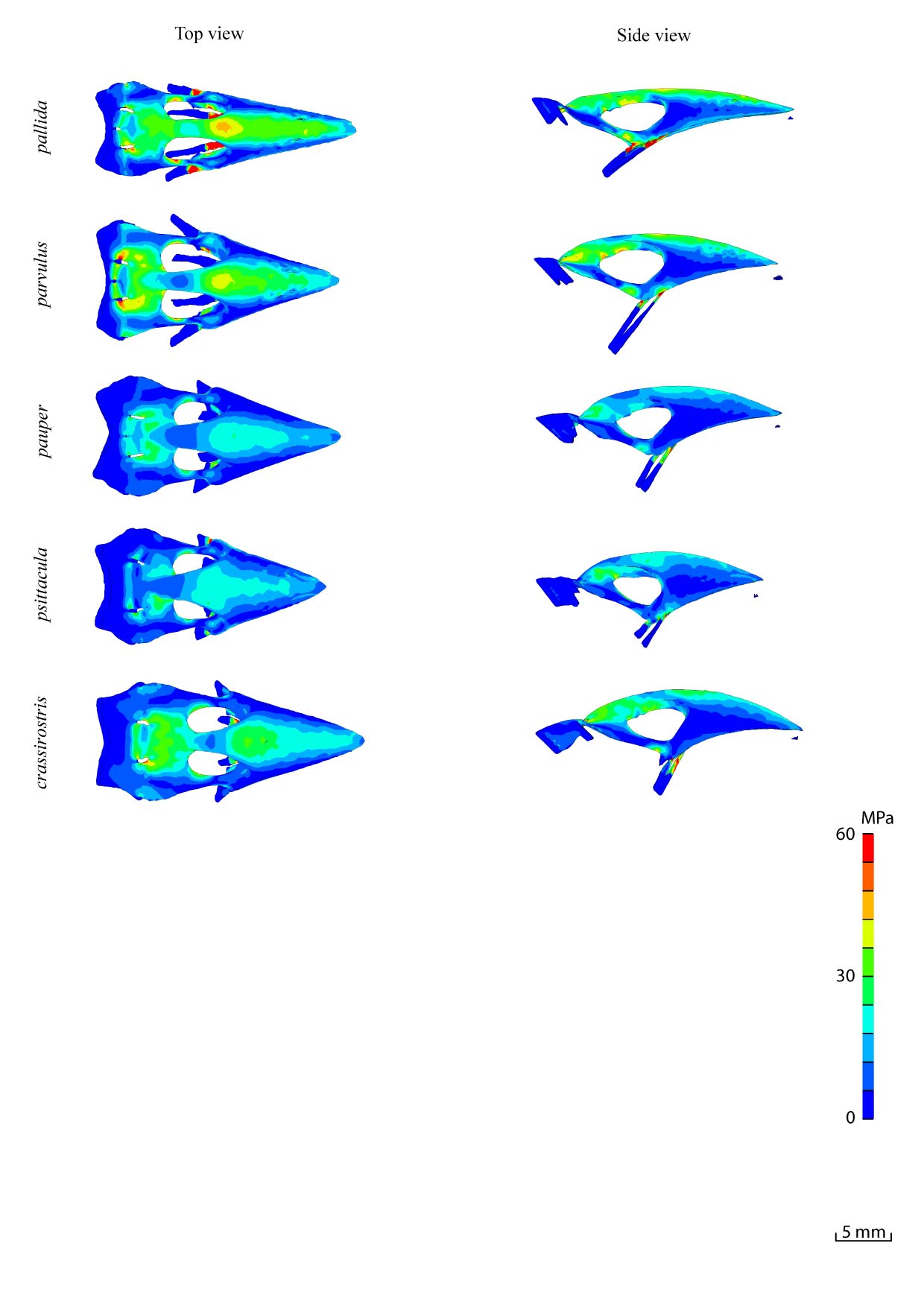


**S5 Fig.:** Top and side view for scaled FE models of upper beak during tip biting for 13 Darwin finches.

Supplement: S5 Fig — (DOCX) [file pone.0129479.s005.docx]
